# The association between nutritive, non-nutritive sucking habits and primary dental occlusion

**DOI:** 10.1186/s12903-018-0610-7

**Published:** 2018-08-22

**Authors:** Hiu Tung Bonnie Ling, Fung Hou Kumoi Mineaki Howard Sum, Linkun Zhang, Cindy Po Wan Yeung, Kar Yan Li, Hai Ming Wong, Yanqi Yang

**Affiliations:** 1 Department of Paediatric Dentistry and Orthodontics, Faculty of Dentistry, Prince Philip Dental Hospital, The University of Hong Kong, 2/F, 34 Hospital Road, Sai Ying Pun, Hong Kong SAR, China; 2 Department of Orthodontics, Tianjin Stomatological Hospital of Nankai University, 75 Dagu Road, Tianjin, China; 3 Translational Research Laboratory, Faculty of Dentistry, Prince Philip Dental Hospital, The University of Hong Kong, Room 7A26, 7/F, 34 Hospital Road, Sai Ying Pun, Hong Kong SAR, China

**Keywords:** Nutritive sucking habit, Non-nutritive sucking habits, Primary dental occlusion

## Abstract

**Background:**

The development of primary dentition can be affected by oral sucking habits. Therefore, this study aims to investigate the association of nutritive and non-nutritive sucking habits with primary dentition development.

**Methods:**

One thousand one hundred and fourteen children aged 2 to 5 years old in Hong Kong were recruited in a cross-sectional study. Information on their nutritive (e.g. breastfeeding and bottle feeding) and non-nutritive sucking habits (e.g. pacifier use and thumb/digit sucking) was collected via questionnaires. The children’s primary occlusions were examined in three dimensions.

**Results:**

Children who were breastfed for more than 6 months had a lower proportion of daily pacifier use (*p* < 0.05). Children who used pacifiers daily had a higher proportion of thumb/digit sucking (*p* < 0.05). Children who used pacifiers daily for more than one year had higher chances of developing an anterior open bite (*p* < 0.05) and a reduced overbite (*p* < 0.05). Those exhibiting daily thumb/digit sucking for more than one year had higher chances of developing Class II incisor and Class II canine relationships, an increased overjet and anterior open bite (*p* < 0.05).

**Conclusion:**

Pure breastfeeding for more than 6 months is inversely associated with daily pacifier use and daily pacifier use is positively associated with daily thumb/digit sucking. Children with more than one year of daily pacifier use and thumb/digit sucking have higher chances of developing abnormal dental relationships in the sagittal (i.e. Class II incisor and Class II canine relationships and increased overjet) and vertical (i.e. anterior open bite) dimensions, respectively.

## Background

Primary dentition is the foundation for the development of permanent dentition, in terms of determining space and occlusion for future developing teeth. Malocclusion is a developmental disorder of the maxillofacial system that results from genetic and environmental factors and affects the jaw, tongue and facial soft tissues [1]. As sucking habits are variable environmental factors, knowledge of how such behaviour contributes to or prevents malocclusion can help determine better options for children’s oral health care. Oral sucking habits, such as breastfeeding and bottle sucking, can be categorised as nutritive habits, which are for feeding children, and non-nutritive habits, such as thumb sucking, finger sucking or pacifier use, which are often used to calm and comfort infants [2]. The calming effects have also been used to provide pain relief during minor procedures such as immunization [3]. Apart from the calming effects and providing a sense of security, pacifier use has been found to be associated with protection of sudden infant death syndrome [3–5].

Breastfeeding is a nutritive sucking habit that has been found to have general, immunological, nutritional and oral benefits for the child [6]. The World Health Organization (WHO) recommends exclusive breastfeeding for the first 6 months of life, with some breastfeeding up to 2 years of age [1, 7]. Our recent study showed that pure breastfeeding is associated with reduced chances of developing abnormal primary dentition, such as lower chances of having a Class II incisal relationship and increased overjet. We also found that children with pure breastfeeding for more than 6 months have wider intercanine and intermolar widths [8].

Sucking is a natural instinct and is a baby’s earliest coordinated muscular activity. The action of breastfeeding uses intensive muscular activity and benefits oral motor development [9, 10]. This repetitive action increases muscle tone and promotes correct development, thus ensuring correct oral function [11]. Other oral habits, such as pacifier use or bottle feeding, produce different functional stimuli [12]. It has been found that those who use pacifiers for more than 6 months and those who bottle feed for over 1 year score lower on masticatory function assessments [12].

When an infant does not breastfeed sufficiently, they may develop other types of sucking habits [13]. Some infants adopt non-nutritive sucking habits to cope with frustration, decreased sense of security or an urge for contact [14]. Certain studies have investigated the association between breastfeeding and non-nutritive sucking habits (i.e. pacifier use and thumb sucking) [2, 15–17]. These studies have reported that breastfeeding is associated with lower chances of pacifier use. However, few have focused on the duration or the frequency of these habits [2, 15, 17]. This is rather important from a dental point of view, because the frequency and magnitude of force are crucial for occlusion development, which may lead to malocclusion in the primary dentition. No study has looked into whether or how non-nutritive sucking habits are interrelated.

Knowing the beneficial oral effects of breastfeeding versus bottle feeding [8], it is also worthwhile to know the effects of non-nutritive oral habits. In contrast to calming and comforting infants, pacifier use is reported to have some unfavourable oral effects. If pacifiers are given to infants when they are learning to suck from their mothers’ breasts in the early postpartum period, the use of pacifiers may interfere with proper sucking and cause nipple confusion [18]. Studies have found pacifier use to be associated with an increased prevalence of oral candidiasis, a type of fungal infection [19–22]. Several studies also show that non-nutritive sucking habits are associated with the development of malocclusion in the primary dentition [1, 12, 18]. Nevertheless, the majority of the existing studies do not address the effects of the duration or frequency of non-nutritive sucking habits. However, it is important for dentists and parents to know the frequency and duration of the force required to affect occlusion.

Therefore, the present study aims to (1) determine the associations between nutritive and non-nutritive sucking habits; (2) assess the interrelation between different non-nutritive sucking habits, pacifier use and thumb/digit sucking; and (3) investigate the relationships between various non-nutritive sucking habits and occlusion in the primary dentition. To be representative of having a habit, the frequency and duration of the non-nutritive sucking habits will be emphasised when performing the analyses.

## Methods

### Samples

A total of 10 kindergartens from different districts of Hong Kong participated in our study. This cross-sectional study was carried out with ethics board approval (HKU/HA HKW IRB: UW12–334) and parental consent forms were collected before the examinations. One thousand one hundred and fourteen children aged 2 to 5 years old participated in the survey. Among the one thousand one hundred and fourteen children, only eight hundred and fifty-one children took part in the oral examination and also completed all the questions in the questionnaires.

Fewer participants were recruited for the oral examinations compared to the surveys, as some did not return consent forms to undergo oral examination, refused examination or were uncooperative upon examination. To maintain the integrity of the study results, participants with severe skeletal discrepancy, with cleft lip or palate, or who were non-Asian were excluded. Only children with primary dentition were included in this study.

In a previous similar study, the probability of the event (Class II canine relationship and increased overjet) is around 20% for those without pacifier/digit-sucking habit and the allocation ratio of having pacifier/digit-sucking habit: no habit is about 1.6:1 [23], it was estimated that a sample of 476 individuals would have 90% chance of detecting an odds ratio of 2 with the two-sided significance level setting at 0.05.

Given this sample size determinations and assuming 20% possible non-responses and loses, the final study population had to be at least 595. As the study recruited 851 subjects who participated in both questionnaires and oral examinations, the sample size was sufficient.

### Data collection

The questionnaires were completed by the children’s parents or guardians and collected information regarding the frequencies and durations of the children’s nutritive and non-nutritive sucking habits. After collecting the questionnaires, those who had left certain sections incomplete were contacted by telephone.

One examiner who had more than 5 years of orthodontic training performed all of the oral examinations throughout the study. The examiner performed calibration with another orthodontist before the study. The examination was carried out in the kindergartens with the children in the lying-down position. The equipment used included oral mirrors, probes and rulers. The children’s dental arch relationships were examined in three dimensions (sagittal, vertical and transverse) as listed in Table 1. Duplicated data were collected for 6.23% of the subjects to assess the intra-examiner reliability.

**Table 1:** Oral examination criteria of the children’s three-dimensional dental arch relationships

Sagittal	Vertical	Transverse
**Incisal relationship**- Classified into three categories: Class I, the lower incisor edges occlude with or lie immediately below cingulum plateau of the upper central incisors; Class II, the lower incisor edges lie posterior to the cingulum plateau of the upper incisors; Class III, the lower incisor edges lie anterior to the cingulum plateau of the upper incisors. The overjet is reduced or reversed [44].	**Overbite**- Coverage of the mandibular incisor by the most protruded fully erupted maxillary incisor and recorded as < 1/2 or ≥ 1/2 [11].	**Intermolar width**- Distance between mesiobuccal cusp tips of the maxillary second primary molars [40].
**Canine relationship**- Classified into three categories: Class I, the tip of the maxillary primary canine tooth is in the same vertical plane as the distal surface of the mandibular primary canine; Class II, the tip of the maxillary primary canine tooth is mesial to the distal surface of the mandibular primary canine; Class III, the tip of the maxillary primary canine is distal to the distal surface of the mandibular primary canine [11].	**Anterior openbite**- When there are no vertical contacts between upper and lower incisal edges [9].	**Intercanine width**- Distance from cusp tip to cusp tip of the maxillary primary canines [40].
**Molar relationship** - Classified into three categories: Flush terminal, where the distal surfaces of the upper and lower second primary molars are in the same vertical plane in a centric occlusion; Distal step, where the distal surfaces of the lower primary second molar are in a posterior relationship to the distal surface of the upper second molars in centric occlusion; Mesial step, the distal surfaces of the lower primary second molar are in an anterior relationship to the distal surface of the upper second molars in centric occlusion [11].		**Posterior crossbite**- Recorded when one or more of the maxillary primary canines or molars occluded lingual to the buccal cusps of the opposing mandibular teeth [11].
**Anterior crossbite** – It was recorded when one or more of the maxillary incisors occluded lingual to the mandibular incisors [45].		
**Overjet**- Measured from the palatal surface of the mesial corner of the most protruded fully erupted maxillary incisor to the labial surface of the corresponding mandibular incisor [11]. The degree of overjet was recorded in millimeters. In this study, an overjet of greater than 3.5 mm was considered an increased overjet.		

### Statistical analysis

The associations between different oral habits and their relationships with occlusion were analysed by multinomial logistic regression, logistic regression and multi-way analysis of variance (ANOVA) using the Statistical Package for the Social Sciences (SPSS) (IBM) version 20.0.

Multinomial logistic regressions were used to investigate the associations between the categorical variables, such as the primary incisal, canine and molar relationships with different sucking habits.

Multivariable logistic regression models were used to investigate the associations between the binary outcome variables, such as the associations between the frequencies of non-nutritive sucking habits and breastfeeding durations, between the different non-nutritive sucking habits and between different non-nutritive sucking habits and overjet, anterior crossbite, open bite, overbite and posterior crossbite.

Multi-way ANOVA using the Bonferroni correction of pairwise comparisons was used to compare the mean intercanine and intermolar widths in children exhibiting different non-nutritive sucking habits.

The significance level was set at *p* < 0.05.

## Results

### Sample characteristics and measurement error

The survey included 1114 children aged 2 to 5 years old. The boy (*n* = 609, 54.7%) to girl (*n* = 500, 44.9%) ratio was 1.22. Five children’s parents did not answer the question on the children’s gender.

Regarding the intra-examiner reliability, the Cohen’s kappa coefficients ranged from 0.70 to 1.00 and the Interclass Correlation Coefficient (ICC) ranged from 0.89 to 0.98, indicating that the categorical data were in substantial to perfect agreement and the continuous data had excellent reproducibility [24, 25].

### Correlations between nutritive and non-nutritive sucking habits

Among the 1114 children who had participated in the survey, 80 participants did not provide complete answers to questions on duration of breastfeeding, frequency of pacifier use or frequency of thumb/digit sucking. Therefore, only 1034 children were included in the analysis (Tables 2 and 3).

**Table 2:** Association between the duration of breastfeeding and frequency of non-nutritive sucking habits (*n* = 1034)

			Daily pacifier vs non daily pacifier			Daily thumb/digit sucking vs non daily thumb/digit sucking		
	n	%	OR	95% CI	*p*-value	OR	95% CI	*p*-value
Duration of breastfeeding					0.000*			0.143
> 6 months	**246**	**23.8**	**0.412**	**0.259–0.655**	0.000*	0.599	0.318-1.128	0.112
0-6 months	471	45.6	0.840	0.598–1.180	0.134	1.042	0.645–1.683	0.867
Never	317	30.7	1	–	–	1	–	–

*OR* odds ratio, *CI* confidence interval

^a^ Adjusted for background information (age and gender)

* *p* < 0.05

Boldface data are variables with *p* < 0.05 or OR (95%CI) < 1

**Table 3:** Association between the frequency of pacifier use and frequency of thumb/digit sucking (n = 1034)

Frequency of pacifier use			Daily thumb/digit sucking vs non daily thumb/digit sucking		
	n	%	OR	95% CI	*p* value
Daily pacifier	**212**	**20.5**	**2.136**	**1.112–4.103**	0.023*
Non daily pacifier	822	79.5	1	–	–

*OR* odds ratio, *CI* confidence interval

^a^ Adjusted for background information (age and gender) and duration of pure breastfeeding

* *p* < 0.05

Boldface data are variables with *p* < 0.05 or OR (95%CI) < 1

Significant association was found between the duration of breastfeeding and the frequency of pacifier use (*p* = 0.000). The children who had experienced pure breastfeeding for more than 6 months had a significantly lower chance of daily pacifier use (multinomial logistic regression: *p* = 0.000; adjusted odds ratio [OR] = 0.412, 95% confidence interval [CI] 0.259–0.655). However, no association between the duration of pure breastfeeding and the development of habitual thumb/digit sucking was found (multinomial logistic regression *p* > 0.05). The associations between the duration of breastfeeding and the frequency of pacifier and thumb/digit sucking are presented in Table 2.

### Correlations between non-nutritive sucking habits

Children who used pacifiers daily had significantly higher chances of having daily thumb/digit sucking habits (logistic regression: *p* = 0.023; adjusted OR = 2.136, 95% CI 1.112–4.103) (Table 3).

### Associations between non-nutritive sucking habits and primary dental relationships

Among the 1114 children who had participated in the survey, 851 children took part in the oral examination and also completed all the questions in the questionnaires. Hence, the following tables (Tables 4, 5, 6 and 7) are analysis based on the 851 children.

**Table 4:** Association between frequency of different non-nutritive sucking habits and primary dental relationships in sagittal dimension (*n* = 851)

	Incisor relationship					Canine relationship					Molar relationship					Overjet		
	*p*-value	Class II incisor vs Class I incisor		Class III incisor vs Class I incisor		*p*-value	Class II canine vs Class I canine		Class III canine vs Class I canine		*p*-value	Bilateral Flush vs Bilateral Mesial step		Bilateral Mesial step vs Bilateral Distal step		*p*-value	Overjet > 3.5 mm vs non overjet > 3.5 mm	
		OR	95% CI	OR	95% CI		OR	95% CI	OR	95% CI		OR	95% CI	OR	95% CI		OR	95% CI
Frequency of pacifier use	0.490					0.108					0.168					0.495		
Daily		1.292	0.837–1.996	0.981	0.543–1.773		1.656	1.016–2.699	1.204	0.515–2.813		1.508	0.860–2.647	1.688	0.926–3.077		1.191	0.720–1.970
Non-daily		1	–	1	–		1	–	1	–		1	–	1	–		1	–
Frequency of thumb/digit sucking	0.008*					0.036*					0.622					0.000*		
Daily		**2.237**	**1.290–3.877**	0.765	0.287–2.044		**2.595**	**1.117–6.025**	1.806	0.215–5.478		1.182	0.536–2.607	1.494	0.671–3.325		**2.879**	**1.624–5.101**
Non-daily		1	–	1	–		1	–	1	–		1	–	1	–		**1**	**–**

*OR* odds ratio, *CI* confidence interval

^a^ Adjusted for background information (age and gender) and duration of pure breastfeeding

* *p* < 0.05

Boldface data are variables with *p* < 0.05 or OR (95%CI) < 1

**Table 5:** Association between duration of different non-nutritive sucking habits and primary dental relationships in sagittal dimension (*n* = 851)

	Incisor relationship					Canine relationship					Molar relationship					Overjet		
	*p*-value	Class II incisor vs Class I incisor		Class III incisor vs Class I incisor		*p*-value	Class II canine vs Class I canine		Class III canine vs Class I canine		*p*-value	Bilateral Flush vs Bilateral Mesial step		Bilateral Mesial step vs Bilateral Distal step		*p*-value	Overjet > 3.5 mm vs non overjet > 3.5 mm	
		OR	95% CI	OR	95% CI		OR	95% CI	OR	95% CI		OR	95% CI	OR	95% CI		OR	95% CI
Duration of daily pacifier use	0.483					0.063					0.183					0.448		
> 1 year		1.416	0.881–2.276	0.988	0.520–1.878		1.995	1.162–3.425	1.003	0.371–2.708		1.111	0.588–2.099	1.711	0.896–3.267		1.417	0.826–2.430
< 1 year		1.960	0.683–5.626	1.121	0.229–5.480		1.426	0.371–5.477	2.77	0.432–17.873		4.459	0.836–23.793	3.158	0.511–19.513		1.058	0.291–3.846
Never		1	–	1	–		1	–	1	–		1	–	1	–		1	–
Duration of daily thumb/digit sucking	0.001*					0.005*					0.455					0.000*		
> 1 year		**2.930**	**1.628–5.274**	0.966	0.354–2.635		**3.483**	**1.312–9.245**	0.658	0.073–5.967		1.005	0.415–2.434	1.663	0.715–3.867		**3.603**	**1.987–6.533**
Never		1	–	1	–		1	–	1	–		1	–	1	–		**1**	**–**

*OR* odds ratio, *CI* confidence interval

^a^ Adjusted for background information (age and gender) and different oral habits

* *p* < 0.05

Boldface data are variables with *p* < 0.05 or OR (95%CI) < 1

The group for < 1 year of daily thumb/digit sucking was excluded since the number of subjects were not sufficient to perform the test

**Table 6:** Association between frequency of different non-nutritive sucking habits and primary dental relationships in vertical dimension (n = 851)

	Anterior openbite			Anterior overbite		
	*p*-value	Anterior openbite vs non-anterior openbite		*p*-value	Overbite ≥ ½ vs overbite < ½	
		OR	95% CI		OR	95% CI
Frequency of pacifier use	0.000*			0.004*		
Daily		**10.149**	**3.798–27.122**		**0.555**	**0.373–0.825**
Non-daily		1	–		1	–
Frequency of thumb/digit sucking	0.046*			0.128		
Daily		**3.440**	**1.020–11.597**		0.653	0.377–1.130
Non-daily		1	–		1	–

*OR* odds ratio, *CI* confidence interval

^a^ Adjusted for background information (age and gender) and duration of pure breastfeeding

* *p* < 0.05

Boldface data are variables with *p* < 0.05 or OR (95%CI) < 1

**Table 7:** Association between duration of different non-nutritive sucking habits and primary dental relationships in vertical dimension (*n* = 851)

	Anterior openbite			Anterior overbite		
	*p*-value	Anterior openbite vs non-anterior openbite		*p*-value	Overbite ≥ ½ vs overbite < ½	
		OR	95% CI		OR	95% CI
Duration of daily pacifier use	0.000*			0.045*		
> 1 year		**15.171**	**5.298–43.446**		**0.577**	**0.374–0.890**
< 1 year		0.000	0.000–0.000		0.881	0.302–2.570
Never		1	–		1	–
Duration of daily thumb/digit sucking	0.006*			0.122		
> 1 year		**6.383**	**1.689–24.120**		0.631	0.352–1.131
Never		1	–		1	–

*OR* odds ratio, *CI* confidence interval

^a^ Adjusted for background information (age and gender) and duration of pure breastfeeding

* *p* < 0.05

Boldface data are variables with *p* < 0.05 or OR (95%CI) < 1

The group for < 1 year of daily thumb/digit sucking was excluded since the number of subjects were not sufficient to perform the test

#### Sagittal dimension

In terms of frequency, the children with daily thumb/digit sucking habits had significantly higher chances of developing Class II incisor relationships (multinomial logistic regression: *p* = 0.008; adjusted OR = 2.237, 95% CI 1.290–3.877), Class II canine relationships (multinomial logistic regression: *p* = 0.036; adjusted OR = 2.595, 95% CI 1.117–6.025) and overjets > 3.5 mm (logistic regression: *p* = 0.000; adjusted OR = 2.879, 95% CI 1.624–5.101) than those without daily thumb/digit sucking habits (Table 4). However, the frequency of pacifier use was not associated with primary incisor, primary canine or primary molar relationships (multinomial logistic regression: *p* > 0.05) (Table 4).

Regarding duration, the children who exhibited daily thumb/digit sucking for more than a year had significantly higher chances of developing Class II incisor relationships (multinomial logistic regression: *p* = 0.001; adjusted OR = 2.930, 95% CI 1.628–5.274), Class II canine relationships (multinomial logistic regression: *p* = 0.005; adjusted OR = 3.483, 95% CI 1.312–9.245) and overjets > 3.5 mm (logistic regression: *p* = 0.000; adjusted OR 3.603, 95% CI 1.987–6.533) than those without daily thumb/digit sucking habits (Table 5). Similar to the analysis of frequency, the duration of daily pacifier use was found to have no association with primary incisor, primary canine or primary molar relationships (multinomial logistic regression: *p* > 0.05) (Table 5).

#### Vertical dimension

In terms of frequency, the children who used pacifiers daily had significantly higher chances of developing an anterior open bite (logistic regression: *p* = 0.000; adjusted OR = 10.149, 95% CI 3.798–27.122) and significantly lower chances of developing an overbite greater than half of the lower incisor (logistic regression: *p* = 0.004; adjusted OR = 0.555, 95% CI 0.373–0.825) than those who did not use pacifiers daily. In addition, children with daily thumb/digit sucking habits had significantly higher chances of developing an anterior open bite (logistic regression: *p* = 0.046; adjusted OR = 3.440, 95% CI 1.020–11.597) (Table 6). The frequency of thumb/digit sucking was not associated with the extent of the anterior overbite formed (logistic regression: *p* > 0.05) (Table 6).

The results of the duration analyses were very similar to those of frequency analyses. The children who experienced daily pacifier use for more than 1 year had significantly higher chances of developing an anterior open bite (logistic regression: *p* = 0.000; adjusted OR = 15.171, 95% CI 5.298–43.446) and significantly lower chances of developing an overbite greater than half of the lower incisor (logistic regression: *p* = 0.045; adjusted OR = 0.577, 95% CI 0.340–0.890) than those who never had the habit of daily pacifier use (Table 7). In addition, children who displayed daily thumb/digit sucking for more than 1 year had significantly higher chances of developing an anterior open bite (logistic regression: *p* = 0.006; adjusted OR = 6.383, 95% CI 1.689–24.120) than those who had never displayed daily thumb/digit sucking habits (Table 7).

#### Transverse dimension

The frequency and duration of pacifier use and thumb/digit sucking were neither associated with the development of posterior crossbite (logistic regression: *p* > 0.05) nor the intercanine (ANOVA: *p* > 0.05)/intermolar widths (ANOVA: *p* > 0.05).

## Discussion

First, this study assessed the association between nutritive and non-nutritive sucking habits. It was found that children who were breastfed for more than 6 months had significantly less daily pacifier use. No relationship was found between breastfeeding and thumb/digit sucking. The relationship between breastfeeding and pacifier use is consistent with previous studies [17, 26]. Breastfeeding has been found to use more musculature and facilitates the development of the correct orofacial muscles [11, 12, 27]. With unrestricted breastfeeding infants experience improved safety and satisfaction, and thus no other sucking actions are needed, which leads to less pacifier use [11]. One study found increased digit sucking when breastfeeding lasted less than 6 months [15]. However, another study focusing on the frequency of thumb sucking found no relationship to breastfeeding [18], which is similar to our results. The variability between studies may be due to whether the frequency of thumb sucking was taken into account when the data were analysed.

Second, this study assessed the interrelation between different non-nutritive sucking habits. It was found that more daily pacifier use increased the chances for more thumb/digit sucking habits. Not many studies have focused on the relationship between pacifier use and thumb sucking, apart from a study done in 1977 that found an inverse association between the two habits [28]. A possible explanation as to why pacifier use increased thumb/digit sucking in our study is that adaptation to one habit may increase the urge for and addiction to the sucking sensation. In addition**,** when infants are not sufficiently satisfied by thumb/digit sucking, they may develop other habits to help them to fulfil their needs.

Third, this study investigated the effects of non-nutritive sucking habits on primary dental relationships. In the sagittal dimension, the results of this study agree with previous studies in that thumb sucking is associated with Class II incisor relationships, Class II canine relationships and also increased overjet [29–35]. The higher incidence of increased overjet may be due to proclination of the maxillary incisors and forward displacement of the maxillary base as a result of the pressure of the thumb [36–38]. The overjet may also be worsened by retroclination of the lower incisors due to the lever action of the thumb [39]. The increase in Class II canine relationships may be due to the forward displacement of the anterior maxillary base [35, 39].

Some existing studies have found that pacifier use is associated with increased overjet [11, 40, 41]. Nevertheless, this study did not find any association between pacifier use and the development of primary dentition in the sagittal dimension. The difference may be because the previous studies have not assessed the frequency or duration of pacifier use. Furthermore, most studies have not considered thumb/digit sucking as a confounding factor. In addition, there are different types of pacifiers on the market; different pacifier designs may affect the results.

In the vertical dimension, the results of this study agree with those of existing studies in that thumb/digit sucking and pacifier use are associated with increased open bite [42]. Pressure from the thumb or pacifier hinders the downward growth of the maxillary base and delays the anterior teeth from erupting while the posterior teeth continue to erupt. This results in overeruption of the posterior teeth and the formation of an anterior open bite [11, 33, 35, 36, 43].

Multiple studies have found that non-nutritive sucking habits are associated with smaller maxillary intercanine and intermolar widths and increased posterior crossbite [29, 41]. Nevertheless, this study found no association between non-nutritive sucking habits in the transverse dimension of the primary dentition. These inconsistent findings can be explained by the fact that most of the studies have not accounted for confounding factors, such as age, gender and other non-nutritive sucking habits, in their statistical analyses. Furthermore, many studies have not investigated the frequency or duration of the habits.

The results of this study show that pure breastfeeding for more than 6 months lowered pacifier use, which was associated with less thumb/digit sucking. Together, less pacifier use and less thumb/digit sucking benefited primary dental relationship development in the sagittal and vertical dimensions. These results raise three important points. First, pure breastfeeding for more than 6 months prevents non-nutritive sucking habits. Second, there is a correlation between variable non-nutritive sucking habits. Thus, preventing or breaking a non-nutritive sucking habit may prevent or break others. Third, preventing or breaking non-nutritive sucking habits is important for the development of primary dentition.

This study did have its limitations. The random selection of subjects was difficult, as we needed approval from their schools to take part in the study. Effort was made to spread out the samples across the main territories of Hong Kong. As this is a retrospective study, recall bias is possible. As parents are unable to monitor their children for 24 h each day, there may be an underestimation of thumb/digit sucking habits. Furthermore, the survey questions may have had overlapping response options (e.g. ‘0–6 months’ and ‘6–12 months’). During the oral examinations, the children were all in the lying-down position to prevent them from moving around; however, it may have been more accurate if they were sitting down instead. Finally, it was difficult to assess whether the child had mild skeletal discrepancy without the use of radiographs.

## Conclusion

Breastfeeding for more than 6 months is negatively associated with pacifier use. Pacifier use is positively associated with thumb/digit sucking. Pacifier use and thumb/digit sucking are associated with higher chances of malocclusion in the sagittal (i.e. Class II incisal relationships, Class II canine relationships and increased overjet) and vertical (i.e. anterior open bite) dimensions of the primary dentition.

## Abbreviations

ANOVA: Analysis of variance
ICC: Interclass Correlation Coefficient
OR: Odds ratio
SPSS: Statistical Package for the Social Sciences
WHO: World Health Organization
